# Detecting influenza and emerging avian influenza virus by influenza and pneumonia surveillance systems in a large city in China, 2005 to 2016

**DOI:** 10.1186/s12879-019-4405-5

**Published:** 2019-09-18

**Authors:** Xiaorong Guo, Dong Yang, Ruchun Liu, Yaman Li, Qingqing Hu, Xinrui Ma, Yelan Li, Heng Zhang, Xixing Zhang, Benhua Zhao, Tianmu Chen

**Affiliations:** 1 State Key Laboratory of Molecular Vaccinology and Molecular Diagnostics, School of Public Health, Xiamen University, 4221-117 South Xiang’an Road, Xiang’an District, Xiamen, Fujian Province People’s Republic of China; 2 Changsha Center for Disease Control and Prevention, Changsha, Hunan People’s Republic of China; 3 Division of Public Health, School of Medicine, University of Utah, Salt Lake City, UT USA

**Keywords:** Avian influenza virus, Influenza surveillance system, Pneumonia surveillance system

## Abstract

**Background:**

Detecting avian influenza virus has become an important public health strategy for controlling the emerging infectious disease.

**Methods:**

The HIS (hospital information system) modified influenza surveillance system (ISS) and a newly built pneumonia surveillance system (PSS) were used to monitor the influenza viruses in Changsha City, China. The ISS was used to monitor outpatients in two sentinel hospitals and to detect mild influenza and avian influenza cases, and PSS was used to monitor inpatients in 49 hospitals and to detect severe and death influenza cases.

**Results:**

From 2005 to 2016, there were 3,551,917 outpatients monitored by the ISS system, among whom 126,076 were influenza-like illness (ILI) cases, with the ILI proportion (ILI%) of 3.55%. After the HIS was used, the reported incident cases of ILI and ILI% were increased significantly. From March, 2009 to September, 2016, there were 5,491,560 inpatient cases monitored by the PSS system, among which 362,743 were pneumonia cases, with a proportion of 6.61%. Among pneumonia cases, about 10.55% (38,260/362,743) of cases were severe or death cases. The pneumonia incidence increased each year in the city. Among 15 avian influenza cases reported from January, 2005 to September, 2016, there were 26.7% (4/15) mild cases detected by the HIS-modified ISS system, while 60.0% (9/15) were severe or death cases detected by the PSS system. Two H5N1 severe cases were missed by the ISS system in January, 2009 when the PSS system was not available.

**Conclusions:**

The HIS was able to improve the efficiency of the ISS for monitoring ILI and emerging avian influenza virus. However, the efficiency of the system needs to be verified in a wider area for a longer time span in China.

## Background

In recent years, reports about human cases of infected emerging avian influenza virus has become more and more common [1–4]. Human infected H5N1 virus was first reported in 1997 in Hong Kong, China, and then was spread widely in mainland China [5] as well as many other countries in Southeast Asia [6, 7], West Asia [8, 9], and Africa [10]. Thereafter, human cases infected with emerging influenza viruses were reported in the subsequent years, especially in China. For example, H7N9 was reported in 2013 [11], H5N6 in 2014 [2, 12–15], H10N8 in 2013 [1], and H9N2 in 2013 in Hunan province, China [16]. It is important to detect these emerging viruses in and out of China.

There are currently two main surveillance systems focused on detecting / monitoring the virus in China. One is China’s national sentinel surveillance system for influenza-like illness (ILI) in sentinel hospitals across 31 provinces in mainland China. The sentinel hospitals are accounted for 2.5% of all hospitals in China [17]. The other one is the national pneumonia surveillance system, which was built by the Chinese Center for Disease Control and Prevention (CDC) in 2004. The system is mainly to monitor pneumonia of unknown etiology (PUE) to facilitate timely detection of novel respiratory pathogens, such as severe acute respiratory syndrome (SARS) and avian influenza [18]. The two systems have played significant roles in monitoring the activity of influenza, controlling and preventing emerging avian influenza [17–19].

Traditionally, the number of ILI cases was counted manually by influenza surveillance staff. Therefore, the hospital information system (HIS) has been used for recording and monitoring the outpatients in most hospitals in China [20, 21]. The PUE surveillance system was not (and is still not) used consistently because most cases on community-acquired pneumonia met the PUE criteria, but were not reported to the PUE system [18]. The PUE surveillance system was not sensitive enough to detect the emerging avian influenza virus.

To explore a new way to monitor influenza virus by using HIS and improve the sensitivity of detecting emerging avian influenza virus, we modified the influenza surveillance system (ISS) and built a pneumonia surveillance system (PSS) in Changsha City, China. In this study, we separately reported the roles of the ISS and the PSS systems in detecting influenza and emerging avian influenza virus.

## Methods

### Study area

**Fig. 1:**
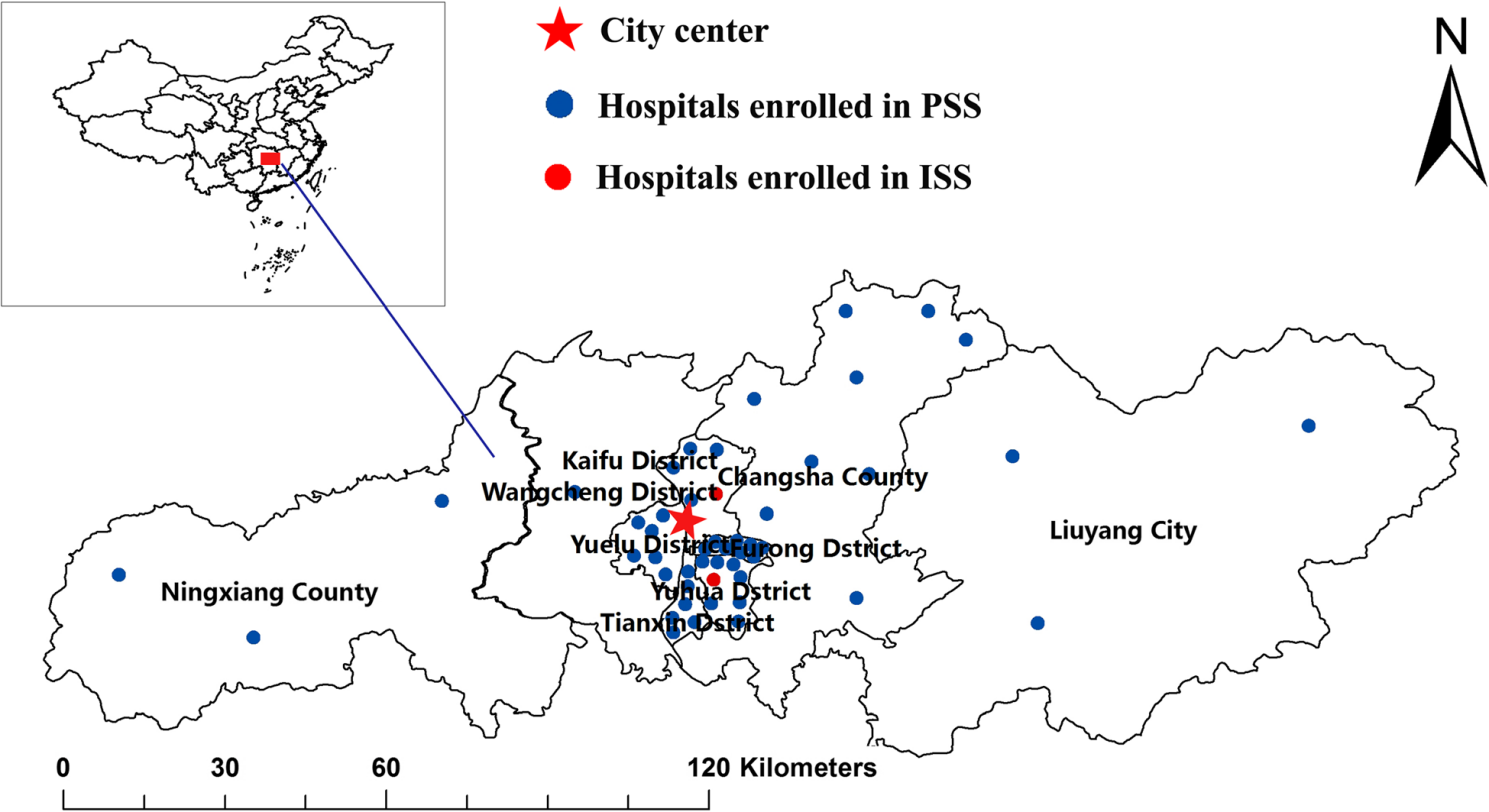
Locations of 49 hospitals in pneumonia surveillance system and 2 hospitals influenza surveillance system in Changsha City, China. The map depicted in this figure was taken from Wikimedia Commons (http://commons.wikimedia.org/wiki/Main_Page). PSS, pneumonia surveillance system; ISS, influenza surveillance system

Changsha (27°51′~ 28°41′ N, 111°53′~ 114°15′ E), a large city with 7.04 million people in central south China, is the capital of Hunan Province. It includes 6 districts, 2 counties, and 1 county-level city. There are a total of 4586 hospitals, clinics, and public health departments all over the city. In this study, there were 49 secondary and tertiary hospitals included into the PSS system and 2 tertiary hospitals into the ISS system, respectively. The two hospitals in ISS system were also included in the PSS. The locations of the selected hospitals were shown in Fig. 1.

### The modified influenza surveillance system

The ISS, based on two sentinel hospitals (hospitals A and B), was set up in Changsha in September 2005. Hospital A, which is located in the south urban area of the city, is a tertiary hospital with more than 2700 staffs and 1839 hospital beds. Hospital B, which is located in the north urban area of the city, is also a tertiary hospital with more than 1700 staffs and 1593 hospital beds. Both hospitals have the administrative department that is in charge of the routine surveillance. Hospital A is a municipally designated hospital for the diagnosis and treatment of tuberculosis.

In 2006 and 2008, the ISS system became a branch of Hunan provincial and national influenza surveillance network respectively. In February, 2012, the HIS was adopted for the surveillance in hospital A, and was modified after June, 2013. The ISS in Changsha underwent five stages (stage 1: week 39, 2005 to week 52, 2005; stage 2: week 1, 2006 to week 52, 2007; stage 3: week 1, 2008 to week 5, 2012; stage 4: week 6, 2012 to week 24, 2013; stage 5: week 25, 2013 to week 41, 2016.). During stage 1 to 3, the two sentinel hospitals registered ILI cases manually in five outpatient departments, including outpatient and emergency departments of respiratory medicine, outpatient and emergency departments of pediatrics, and fever clinic. During stage 4 to 5, hospital B remained the manual surveillance in the same outpatient departments. ILI case was defined as “fever (axillary temperature ≥ 38°C) + cough or sore throat” [22, 23].

Differently, in hospital A, HIS was adopted into the ISS during stage 4 to 5 and was also named as “HIS (stage 1)” and “HIS (stage 2)” respectively. During stage 4, all outpatient departments of the hospital were included in the ISS, and the computer would emerge a popup window by HIS with the question that “ILI or not” if physicians diagnosed one of the 108 influenza-associated diseases based on the International Classification of Diseases 10th Revision (ICD-10). The physician should answer the question to continue the later part to treat the diseases from the patients. But we found that some ILI cases could still be missed probably because of the misunderstanding of the definition of ILI of the physician, especially if the physician was not in the department with the ISS system during stage 1 to 3. Therefore, during stage 5, the question was changed to three options: a) fever (axillary temperature ≥ 38 °C), b) cough, c) sore throat. The procedure of HIS would count the ILI automatically by computing the number of “a) + b)”, “a) + c)”, and “a) + b) + c)”.

**Fig. 2:**
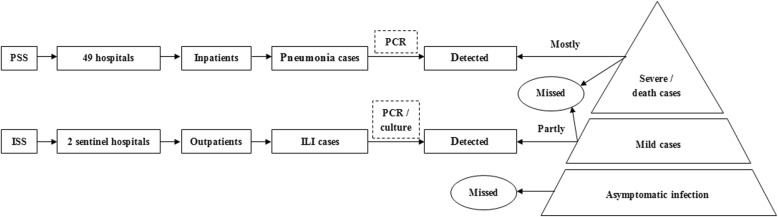
Flowchart of detecting influenza and avian influenza virus by influenza surveillance system and pneumonia surveillance system in Changsha City, China. PSS, pneumonia surveillance system; ISS, influenza surveillance system; ILI, influenza-like illness; PCR, polymerase chain reaction

During the 5 stages, the patients who visited the outpatient departments of the two hospitals and were identified as potential ILI cases, were calculated every week. At least 5–20 throat swab samples of ILI cases per hospital per week were collected for testing the influenza virus by reverse transcription polymerase chain reaction (RT-PCR) and / or cell culture in the laboratory of Changsha CDC. The criteria for including ILI patients who were chosen to collect the samples were: a) the patients were in three days after illness onset date; b) the patients had no history of using antivirals. These sample selection and laboratory surveillance procedures were based on the National Influenza Surveillance Program (2010 edition and 2017 edition) which was announced by the National Health Commission of the People’s Republic of China. This system may monitor influenza and emerging avian influenza cases with mild symptoms or at the early stage of the infection (Fig. 2). Data of the system from week 39, 2005 to week 41, 2016 were collected in our study. Because H1N1pdm was firstly emerged in 2009 [24, 25], the virus was not tested in ISS stages 1 and 2 (Table 1).

**Table 1:** Outcomes during different surveilled stages of influenza surveillance system in Changsha City, China

Sentinel hospitals	Stages	Surveilled patients	ILI	ILI%	Number of tested specimens	Number of positive									
						H3N2	H1N1	H1N1pdm	A (untyped)	B	H5N1	H5N6	H7N9	H9N2	Total
Hospital A	1	8203	188	2.29	30	0	1	NA	0	0	0	NA	NA	0	1
	2	123,731	2100	1.70	721	53	20	NA	0	8	0	NA	NA	0	81
	3	503,835	17,190	3.41	2692	47	31	188	59	64	0	NA	NA	0	389
	4	379,441	10,014	2.64	641	41	0	31	0	12	0	NA	1	0	85
	5	1,163,601	66,779	5.74	3534	178	0	124	1	202	0	1	0	2	508
	Total	2,178,811	96,271	4.42	7618	319	52	343	60	286	0	1	1	2	1064
Hospital B	1	7754	452	5.83	29	0	1	NA	0	0	0	NA	NA	0	1
	2	177,460	2089	1.18	596	50	30	NA	0	1	0	NA	NA	0	81
	3	479,086	13,045	2.72	2626	58	52	171	46	73	0	NA	NA	0	400
	4	174,345	2382	1.37	651	43	0	30	0	8	0	NA	0	0	81
	5	534,461	11,837	2.21	3393	151	0	66	0	172	0	0	0	0	389
	Total	1,373,106	29,805	2.17	7295	302	83	267	46	254	0	0	0	0	952
Total	1	15,957	640	8.12	59	0	2	NA	0	0	0	NA	NA	0	2
	2	301,191	4189	2.87	1317	103	50	NA	0	9	0	NA	NA	0	162
	3	982,921	30,235	6.13	5318	105	83	359	105	137	0	NA	NA	0	789
	4	553,786	12,396	4.01	1292	84	0	61	0	20	0	NA	1	0	166
	5	1,698,062	78,616	7.95	6927	329	0	190	1	374	0	1	0	2	897
	Total	3,551,917	126,076	3.55	14,913	621	135	610	106	540	0	1	1	2	2016

*ILI* influenza-like illness, *NA* not available

### The new pneumonia surveillance system

The PSS was built in Changsha in March, 2009. Pneumonia related inpatient departments in all 49 hospitals (excluding the primary health care centers and private clinics) in Changsha were enrolled into the system. This system monitors pneumonia cases among inpatient population. The public health staff in the surveillance hospitals would count the total number of monitored inpatients, pneumonia cases, severe or death pneumonia cases diagnosed by physicians and then they reported to CDC monthly. When cases were suspected as infected with avian influenza virus by clinicians, the throat swab or lower respiratory tract samples of the suspicious patients (either pneumonia cases, severe or death pneumonia cases) were collected for testing the virus by RT-PCR in the laboratory of Changsha CDC. All the surveillance procedures were performed in every month of each year. However, because H5N6 was first emerged in 2014 and H7N9 in 2013 in China [2, 12, 15, 17], the viruses were not tested from ISS stage 1 to 4, and from ISS stage 1 to 3, respectively (Table 1). The PSS system may monitor influenza and emerging avian influenza cases with severe symptoms or death (Fig. 2). In this study, we collected the data of the system from March, 2009 to September, 2016.

### Statistical methods

The sentinel hospitals A and B are located in the south and north in the same city. We assumed that the outpatients were from the same age group. Therefore, three indicators (*d*_1_, *d*_2_ and *d*_3_) were used to compare the difference between the two hospitals among the five stages. They were the differences of weekly number of monitored outpatients, ILI, and ILI% of the two hospitals, and were expressed as follows:

$$ {d}_1={x}_A-{x}_B $$

$$ {d}_2={y}_A-{y}_B $$

$$ {d}_3={z}_A-{z}_B $$

*x*_*A*_, *x*_*B*_, *y*_*A*_, *y*_*B*_, *z*_*A*_ and *z*_*B*_ refer to weekly number of monitored outpatients of hospital A, weekly number of monitored outpatients of hospital B, weekly ILI of hospital A, weekly ILI of hospital B, weekly ILI% of hospital A, and weekly ILI% of hospital B.

The Analysis of variance (ANOVA) was employed to show the *d*_1_, *d*_2_ and *d*_3_ among the five surveillance stages of the two sentinel hospitals. If there is a statistical significance, the Least Significant Difference (LSD) method will be adopted to conduct the multiple comparisons between any two stages. *P* <  0.05 was considered statistically significant.

## Results

### Influenza surveillance system

From week 39 (from 26th September to 2nd October) in 2005 to week 41 (from10^th^ October to 16th October) in 2016, a total of 3,551,917 outpatients were monitored by the ISS system, among whom 126,076 were ILI cases, with the ILI% of 3.55% (Table 1). From the two sentinel hospitals, the total monitored outpatients numbers were 2,178,811 and 1,373,106, ILI were 96,271 and 29,805, and ILI% were 4.42 and 2.17%, respectively (Table 1).

**Fig. 3:**
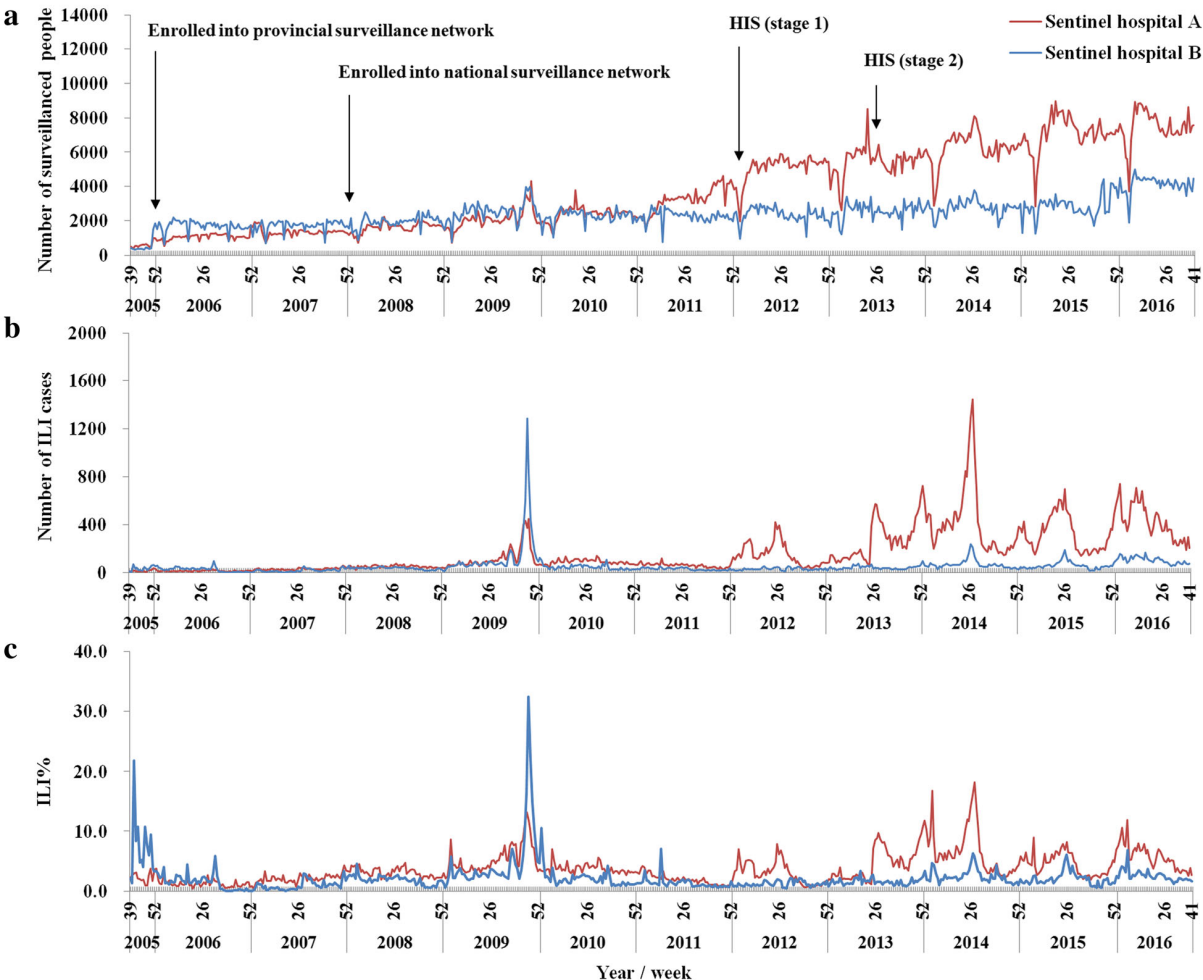
Temporal distributions of weekly monitored outpatients, ILI and ILI% of two sentinel hospitals in Changsha City form week 39, 2005 to week 41, 2016. **a**, temporal distributions of weekly monitored outpatients; **b**, temporal distributions of weekly ILI; **c**, temporal distributions of weekly ILI%

The results of ANOVA showed that the *d*_1_, *d*_2_ and *d*_3_ were significantly different among the five surveillance stages (*P* <  0.001). The results of multiple comparisons by LSD method showed a significant difference in the number of outpatients monitored weekly between hospital A and B between any two stages except between stage 1 and 3. The weekly ILI in hospital A during stage 4 and 5 were shown to be statistically significant compared to hospital B, but no statistical significance during the stages from 1 to 3. The weekly numbers of ILI% in hospital A were shown significance to hospital B during almost all stages except between stage 3 and 4 (Table 2). These results indicated that HIS could improve the efficiency of ISS system in monitoring ILI and ILI% significantly (Fig. 3).

**Table 2:** Multiple comparisons of surveilled patients, ILI and ILI% among different stages between two sentinel hospitals based on Least Significant Difference method

Dependent variables	Stages (I)	Stages (J)	Mean difference (I-J)	*P*
Surveilled patients	Stage 1	Stage 2	548.7(^a^)	0.006
		Stage 3	−83.6	0.667
		Stage 4	− 2856.6(^a^)	< 0.001
		Stage 5	− 3583.7(^a^)	< 0.001
	Stage 2	Stage 1	− 548.7(^a^)	0.006
		Stage 3	− 632.3(^a^)	< 0.001
		Stage 4	− 3405.3(^a^)	< 0.001
		Stage 5	− 4132.4(^a^)	< 0.001
	Stage 3	Stage 1	83.66	0.667
		Stage 2	632.3(^a^)	< 0.001
		Stage 4	− 2773.0(^a^)	< 0.001
		Stage 5	− 3500.1(^a^)	< 0.001
	Stage 4	Stage 1	2856.6(^a^)	< 0.001
		Stage 2	3405.3(^a^)	< 0.001
		Stage 3	2773.0(^a^)	< 0.001
		Stage 5	− 727.1(^a^)	< 0.001
	Stage 5	Stage 1	3583.7(^a^)	< 0.001
		Stage 2	4132.4(^a^)	< 0.001
		Stage 3	3500.1(^a^)	< 0.001
		Stage 4	727.1(^a^)	< 0.001
ILI	Stage 1	Stage 2	−19.0	0.557
		Stage 3	−38.2	0.222
		Stage 4	−126.4(^a^)	< 0.001
		Stage 5	− 334.6(^a^)	< 0.001
	Stage 2	Stage 1	19.0	0.557
		Stage 3	−19.3	0.156
		Stage 4	− 107.4(^a^)	< 0.001
		Stage 5	− 315.7(^a^)	< 0.001
	Stage 3	Stage 1	38.2	0.222
		Stage 2	19.3	0.156
		Stage 4	−88.1(^a^)	< 0.001
		Stage 5	− 296.4(^a^)	< 0.001
	Stage 4	Stage 1	126.4(^a^)	< 0.001
		Stage 2	107.4(^a^)	< 0.001
		Stage 3	88.1(^a^)	< 0.001
		Stage 5	−208.3(^a^)	< 0.001
	Stage 5	Stage 1	334.6(^a^)	< 0.001
		Stage 2	315.7(^a^)	< 0.001
		Stage 3	296.4(^a^)	< 0.001
		Stage 4	208.3(^a^)	< 0.001
ILI%	Stage 1	Stage 2	−5.3(^a^)	< 0.001
		Stage 3	−5.9(^a^)	< 0.001
		Stage 4	−6.1(^a^)	< 0.001
		Stage 5	−8.4(^a^)	< 0.001
	Stage 2	Stage 1	5.3(^a^)	< 0.001
		Stage 3	−0.6(^a^)	0.029
		Stage 4	−0.8(^a^)	0.018
		Stage 5	−3.1(^a^)	< 0.001
	Stage 3	Stage 1	5.9(^a^)	< 0.001
		Stage 2	0.6(^a^)	0.029
		Stage 4	−0.2	0.452
		Stage 5	−2.5(^a^)	< 0.001
	Stage 4	Stage 1	6.1(^a^)	< 0.001
		Stage 2	0.8(^a^)	0.018
		Stage 3	0.2	0.452
		Stage 5	−2.3(^a^)	< 0.001
	Stage 5	Stage 1	8.4(^a^)	< 0.001
		Stage 2	3.1(^a^)	< 0.001
		Stage 3	2.5(^a^)	< 0.001
		Stage 4	2.3(^a^)	< 0.001

^a^ The mean difference is significant at the 0.05 level. ILI, influenza-like illness

**Fig. 4:**
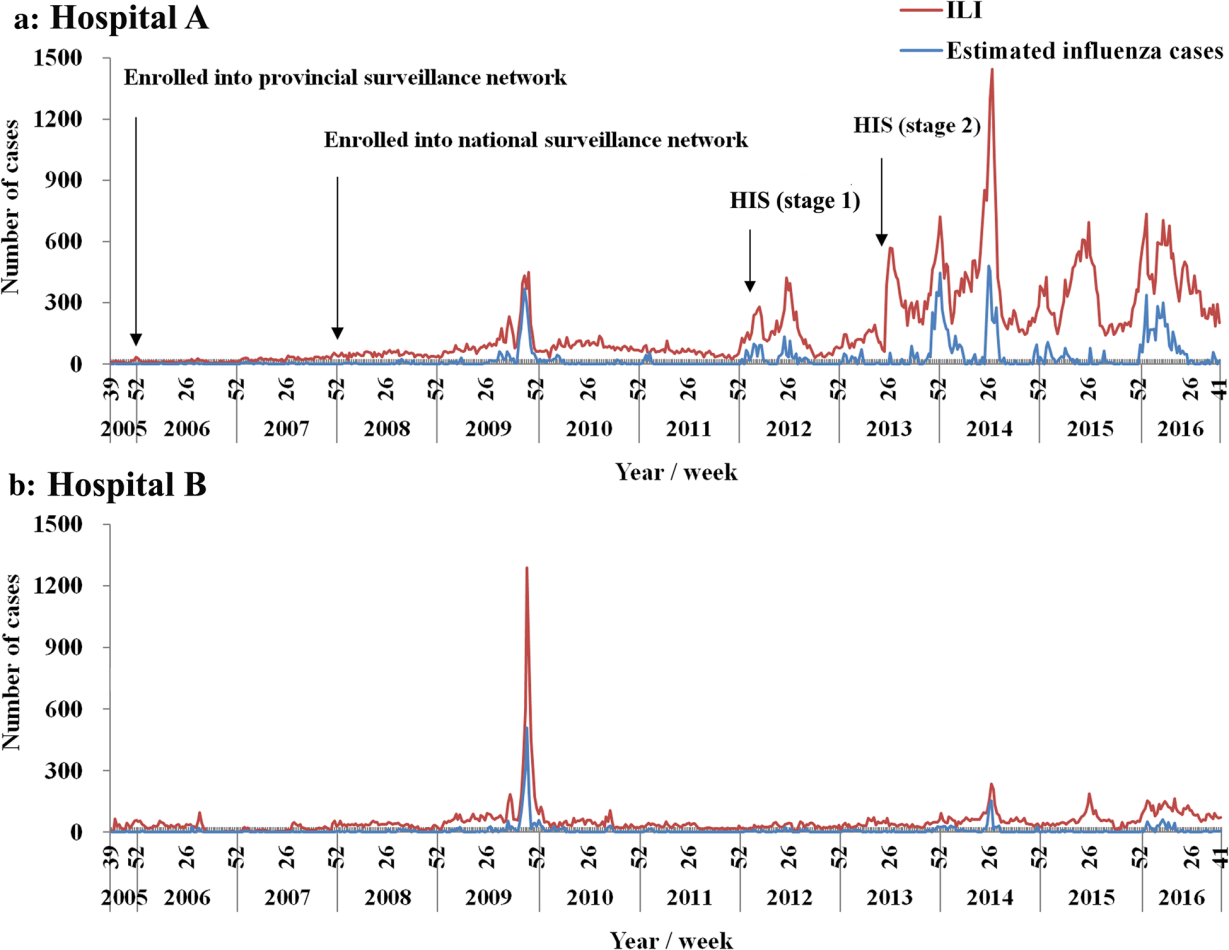
Temporal distributions of weekly ILI and weekly number of estimated influenza cases in two sentinel hospitals in Changsha City form week 39, 2005 to week 41, 2016. **a** temporal distributions of weekly ILI and influenza cases in hospital A; **b** temporal distributions of weekly ILI and influenza cases in hospital B. The estimated influenza cases = the number of ILI cases × the proportion of positive samples

**Fig. 5:**
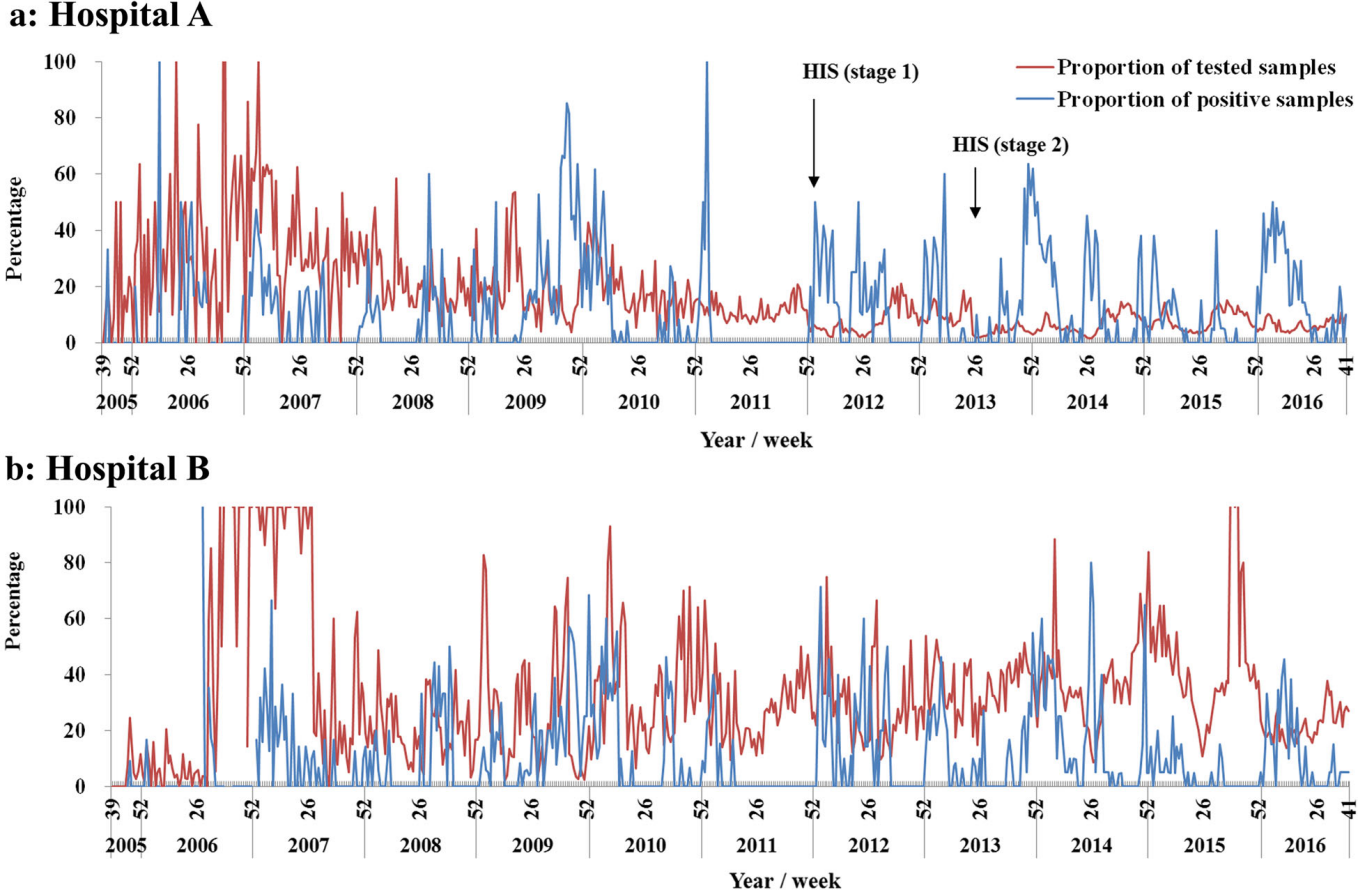
Temporal distributions of weekly proportion of tested samples and weekly proportion of positive samples in two sentinel hospitals in Changsha City, week 39, 2005 to week 41, 2016. **a** Hospital A; **b** Hospital B

**Fig. 6:**
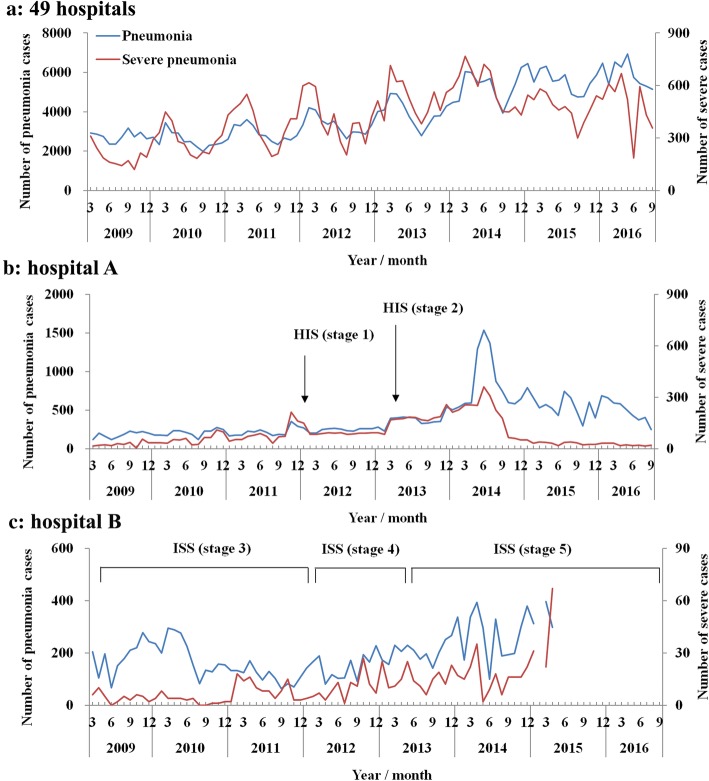
Temporal distributions of pneumonia cases in 49 hospitals and two influenza surveillance sentinel hospitals in Changsha City, March, 2009 to September, 2016. **a** 49 hospitals; **b** hospital A; **c** hospital B

The seasonality of ILI in hospital A matched the activity of influenza virus better than hospital B after the use of HIS (Fig. 4). Two ILI peaks were almost recorded in each year from the ISS system during stages 4 and 5 in hospital A. One peak was recorded in the alternation of winter to spring, and the other one was in summer (Fig. 4). However, the seasonality of ILI was not shown in hospital B. Because of the HIS, the proportion of tested sample descended in hospital A. However, the proportion of positive samples almost had same tendency in the two hospitals (Fig. 5). The inpatient cases on pneumonia ascended after “HIS (stage 1)” in hospital A (Fig. 6).

There was a total of 14,913 throat swabs collected from the two hospitals during the five surveillance stages, among which 2016 were tested positive to influenza or avian influenza virus. Among the positive results, there were 621 with H3N2, 135 with H1N1 (seasonal influenza virus), 610 with H1N1pdm (influenza A / H1N1 from 2009), 106 with untyped influenza A, 540 with B, 1 with H5N6, 1 with H7N9, and 2 with H9N2 virus. The numbers of influenza virus from the two hospitals were similar during the five stages. However, the outcomes of monitoring avian influenza virus were different between the two hospitals. In stage 4, one mild H7N9 case was captured in hospital A when the HIS was adopted at HIS (stage 1). In stage 5, one mild H5N6 case and two mild H7N9 cases were captured in hospital A when the HIS was adopted at HIS (stage 2) (Table 1).

### Pneumonia surveillance system

From March, 2009 to September, 2016, there were 5,491,560 inpatients monitored by the PSS system and 362,743 pneumonia cases, with the rate of 6.61%. About 10.55% (38,260/362,743) of the cases reported on pneumonia were severe or death cases. The incidence of pneumonia increased each year in Changsha (Fig. 6). The seasonality of pneumonia was observed in spring (Fig. 6a).

We collected 3401 throat swab or lower respiratory tract samples, among which 2094 were tested positive to influenza or avian influenza virus. Among the positive results, 78 were H3N2, 17 were seasonal H1N1, 1871 were H1N1pdm, 103 were untyped influenza A, 16 were B, 1 was H5N6, and 8 were H7N9 virus. The activity of H7N9 virus and that of seasonal viruses especially H1N1pdm were observed in 2014 (Table 3).

**Table 3:** Outcomes of pneumonia surveillance system in Changsha City, China

Year	Surveilled patients	Number of pneumonia cases	Number of severe / death cases	Number of tested specimens	Number of positive									
					H3N2	H1N1	H1N1pdm	A (untyped)	B	H5N1	H5N6	H7N9	H9N2	Total
2009	394,683	27,388	1892	2944	69	17	1794	102	0	0	0	0	0	1982
2010	650,244	30,503	3431	136	1	0	9	1	6	0	0	0	0	17
2011	748,361	34,602	4537	142	0	0	29	0	5	0	0	0	0	34
2012	820,482	39,831	4890	10	3	0	0	0	0	0	0	0	0	3
2013	883,769	47,283	6250	29	0	0	0	0	0	0	0	1	0	1
2014	798,792	62,726	7041	81	5	0	21	0	4	0	0	7	0	37
2015	672,289	67,205	5765	16	0	0	0	0	0	0	0	0	0	0
2016	522,940	53,205	4454	43	0	0	18	0	1	0	1	0	0	20
Total	5,491,560	362,743	38,260	3401	78	17	1871	103	16	0	1	8	0	2094

### Emerging avian influenza cases

There were 15 avian influenza cases reported from January, 2005 to September, 2016 (Table 4). Four cases were mild ones that were detected by the ISS system. Nine cases were severe or death cases that were detected by the PSS system. Two H5N1 severe cases were missed by both systems in January, 2009 when the PSS system was not available. Two H5N6 cases, of which one was mild case reported by the ISS system in 2014 and the other was severe case reported by the PSS system in 2016, were detected in Changsha. Nine H7N9 cases were reported by the two systems. One of them was mild case and was detected by the ISS system in 2013 and the other eight were severe / death cases detected by the PSS system in 2014. Two H9N2 cases were mild cases and were detected by the PSS system in 2016.

**Table 4:** Avian influenza cases detected by different ways in Changsha City, China

Avian influenza virus	Category of cases	Detected by ISS system	Detected by PSS system	Other ways	Total
H5N1	Mild cases	0	0	0	0
	Severe / death cases	0	0	2	2
H5N6	Mild cases	1	0	0	1
	Severe / death cases	0	1	0	1
H7N9	Mild cases	1	0	0	1
	Severe / death cases	0	8	0	8
H9N2	Mild cases	2	0	0	2
	Severe / death cases	0	0	0	0
Total	Mild cases	4	0	0	4
	Severe / death cases	0	9	2	11

*ISS* influenza surveillance system, *PSS* pneumonia surveillance system

## Discussion

The avian influenza viruses have spread to many countries including China, Thailand [26], Vietnam [27], Cambodia [28], Turkey [8], the Republic of Azerbaijan [9], Indonesia [29], and Egypt [10, 30]. To control the transmission of the disease, surveillance systems with high sensitivity and specificity need to be created. The surveillance system is commonly used for monitoring ILI for detecting avian influenza virus [30]. In Egypt, clinicians refer all persons with ILI and < 2-week history of poultry contact to one of the Chest and Fever hospitals throughout the country. The respiratory samples of ILI cases are collected to test influenza virus. Daily respiratory samples are also collected from 2 ILI cases and from all patients admitted with severe acute respiratory infection (defined as hospitalization occurring within 2 weeks of onset of fever and cough) in 8 sentinel sites [30].

In Changsha City, the ISS and PSS are different with the surveillance systems in Egypt. The ISS focuses on detecting mild cases while the PSS focuses on severe or death cases infected with influenza and emerging avian influenza viruses (Fig. 2). Therefore, the two systems together could monitor the viruses with different symptoms. The PSS, which covered by all secondary and tertiary hospitals in the city, is built primarily to monitor pneumonia cases. Therefore, the PSS could provide us with the data to estimate the burden of pneumonia and the pathogen spectrum of pneumonia including influenza and emerging avian influenza viruses.

According to our results, we found that the efficiency for monitoring ILI and emerging avian influenza virus was improved after the HIS was used in hospital A. The differences of ILI were significant between stages 1 to 3 and stages 4 to 5, and between hospital A and hospital B after the HIS was used. All the mild emerging avian influenza cases (H5N6, H7N9, H9N2) were detected in the hospital A where the HIS was adopted. These results would encourage a higher coverage of the HIS and the quality of the influenza surveillance in China, to reduce the repetitive work of doctors and avoid bias from the manual counting. Moreover, the probability of finding out influenza and avian influenza virus would be higher than the existing surveillance system because more ILI cases would be recorded by HIS.

Because the ISS only focuses on the mild cases or the early stage of the infection, the surveillance of severe influenza or avian influenza cases is dependent on the PSS. The PSS is a system built in Changsha City and covers all the hospitals that have inpatient departments. Because the PSS focuses on pneumonia cases instead of PUE cases, the system might be more sensitive in detecting influenza virus and emerging avian influenza virus. The PSS has not only given us a way to estimate the disease burden of pneumonia cases in the city, but also has played an important role in controlling and preventing seasonal influenza each year, the pandemic of influenza A (H1N1) in 2009, and the emerging avian influenza such as H5N6 and H7N9 by detecting the severe cases of the infection with these viruses.

Co-infection of seasonal and avian influenza viruses was commonly observed [31]. However, the co-infection was not observed by the ISS in our study. But the activity of H7N9 virus and that of seasonal viruses especially H1N1pdm were observed by the PSS in 2014. This difference might be due to the limited number of reported avian influenza cases in the PSS.

### Limitations

This study has several limitations. Firstly, in the surveillance procedure of the two systems, the tested samples were not chosen randomly, which might affect the surveillance outcomes and thus might impact our findings. Secondly, although the two systems have run stably for a long time and covered a large population, the number of avian influenza cases was limited. It still needs a longer time and a larger population to assess the sensitivity and specificity of the systems. In addition, the ISS system only covers two sentinel hospitals, and the coverage of the system should be improved. The missing PSS data in hospital B from late 2015 to 2016 might underestimate the ability of the PSS system in detecting the viruses. Limited by the small number of reported avian influenza cases in PSS, the co-infection of seasonal and avian influenza viruses might not be observed apparently. Our findings also showed that the number of severe pneumonia cases decreased significantly in late 2014 in Hospital A (Fig. 6). The reason for the decrease remains unclear, but the decrease might slightly affect the results of detecting influenza cases.

## Conclusions

The HIS seems to be able to improve the efficiency of the ISS for monitoring ILI and emerging avian influenza virus. Although the PSS could monitor some emerging avian influenza viruses, the efficiency of the system needs to be verified in a wider area and in a longer time span in China.

## Data Availability

The datasets used and/or analysed during the current study available from the corresponding author on reasonable request.

## Abbreviations

ANOVA: Analysis of variance
CDC: Center for Disease Control and Prevention
HIS: Hospital information system
ICD-10: International Classification of Diseases 10th Revision
ILI: Influenza-like illness
ISS: Influenza surveillance system
LSD: Least Significant Difference
PSS: Pneumonia surveillance system
PUE: Pneumonia of unknown etiology
RT-PCR: Reverse transcription polymerase chain reaction
SARS: Severe acute respiratory syndrome
